# Risk of stroke-associated pneumonia during hospitalization: predictive ability of combined A^2^DS^2^ score and hyperglycemia

**DOI:** 10.1186/s12883-019-1497-x

**Published:** 2019-11-25

**Authors:** Yang Li, Yu Zhang, Liansheng Ma, Xiaoyuan Niu, Junsen Chang

**Affiliations:** Department of Neurology, the First Hospital of Shanxi Medical University, No. 58 Jiefang South Road, Yingze District, Taiyuan, 030000 China

**Keywords:** Stroke-associated pneumonia, A^2^DS^2^ score, Fasting hyperglycemia, Predictive value

## Abstract

**Background:**

Stroke-associated pneumonia (SAP) is a common complication of cerebrovascular disease. The A^2^DS^2^ score has been used to predict the risk of SAP. However, hyperglycemia is not included in this scale. The purpose of the present study was to explore whether the A^2^DS^2^ scoring system and hyperglycemia could predict the risk of SAP more effectively than the conventional A^2^DS^2^ scale.

**Methods:**

This retrospective study enrolled 2552 patients with acute ischemic stroke. The A^2^DS^2^ scores, fasting blood glucose level and blood glucose level on admission were collected. Regression analysis was used to identify the independent risk factors of SAP. ROC curve analysis was used to evaluate the specificity and sensitivity of the combined A^2^DS^2^ score and fasting hyperglycemia for predicting SAP.

**Results:**

Fasting hyperglycemia was an independent risk factor for SAP (OR = 2.95; 95% confidence interval: 2.11–4.12; *P <* 0.001). The area under curve of the combined A^2^DS^2^ score and fasting hyperglycemia was significantly higher than that of the A^2^DS^2^ score alone (0.814 vs. 0.793; *P* = 0.020).

**Conclusion:**

Fasting hyperglycemia is an independent risk factor for predicting SAP. Compared with the A2DS2 score, the modified A2DS2 score (combined A2DS2 score and fasting hyperglycemia) is more effective in predicting the risk of SAP.

## Introduction

Pneumonia is a common critical complication following acute ischemic stroke, and the risk factors include senior age, severe basic diseases, and long duration of hospitalization. Pneumonia increases the duration and expense of hospitalization, and has well been associated with high mortality [1]. Therefore, the early identification and appropriate treatment of post-stroke pneumonia should be highlighted to improve the clinical prognosis.

Stroke-associated pneumonia (SAP) refers to pneumonia occurring during the first seven days after stroke onset in non-ventilated patients [2]. Its incidence has been reported to be approximately 2.3–44% [3–6] .Various risk factors of SAP have been identified, such as male gender, elderly age, dysphagia, severe stroke and disturbance of consciousness. The A^2^DS^2^ scoring system, which was proposed by Hoffmann et al., is a simple and effective evaluation tool for predicting the risk of SAP during hospitalization [7]. This has been verified in China [8–11], France [3], Spain [12], the United Kingdom [13], and Denmark [14]. The A^2^DS^2^ assessment can easily be completed on admission. However, it is still not widely used in clinical practice.

Several studies have indicated that hyperglycemia may also be a risk factor for SAP [6, 14, 15] . Hyperglycemia is not included in the A^2^DS^2^ scoring system [7], and relevant evidence has shown that diabetes is not an independent risk factor for SAP. However, these findings do not contradict the hypothesis that hyperglycemia is a risk factor for SAP, since the blood glucose level of patients with well-controlled diabetes can be normal. Blood glucose level is one of the routine clinical indicators for patients with acute cerebrovascular disease. The value of the combined A^2^DS^2^ score and hyperglycemia for predicting SAP remains unclear.

The purpose of the present study was to investigate the predictive ability of the combined A^2^DS^2^ score and hyperglycemia for the risk of SAP in patients with acute ischemic stroke during hospitalization.

## Materials and methods

### Patients

This retrospective study focused on patients with acute ischemic stroke hospitalized in the First Hospital of Shanxi Medical University from January 2012 to December 2016. Inclusion criteria: (a) magnetic resonance imaging (MRI) revealing an acute cerebral infarction; (b) the patient was admitted within seven days after onset of stroke; (c) random blood glucose level on admission and fasting blood glucose level at the next morning after admission are available. Exclusion criteria: (a) transient ischemic attack (TIA), (b) patients who were discharged or died within three days after onset, (c) patients with pre-existing pneumonia before admission, (d) the lack of more than one of the A^2^DS^2^ scoring items, or (e) mechanical ventilation.

The A^2^DS^2^ scoring system comprised of the following: (1) 1 point for elderly age (≥75 years old); (2) 1 point for male gender; (3) 1 point for atrial fibrillation; (4) 2 points for dysphagia; (5) 3 points for an National Institute of Health stroke scale (NIHSS) score within 5–15 points; (6) 5 points for an NIHSS score > 16 points.

### Data collection

Demographic data (age and gender) were collected. Previous and present medical history (history of atrial fibrillation, dysphagia and diabetes) were reviewed. An electrocardiogram was performed for all patients, and atrial fibrillation was recorded. Our department belongs to the stroke unit and has a professional rehabilitation doctor. At admission, all patients were assessed by rehabilitation physicians based on the Kubota Drinking Test which was conducted by Wata Junfu of Japan [16] for dysphagia and severity. The method of examination is that the patient sits upright and swallows 30 ml of warm water as soon as possible, and observes whether there is cough or not and the time of drinking water in order of times after drinking. The results were divided into five levels: Grade 1, can finish drinking at one time without choking; Grade 2, need more than two swallowing to finish drinking water, but not accompanied by hoarseness or coughing; Grade 3, only need one swallowing movement to finish drinking water, but the voice of edition friends hoarseness or choking cough; Grade 4, need more than two swallowing to finish drinking water, hoarseness or coughing; Grade 5, coughing constantly during swallowing, it is difficult to drink all 30 ml of water. Grade 1 and 2 patients can eat freely. Grade 3 and 4 patients are given dietary guidance by rehabilitation physicians, such as adding thickeners. Grade 5 patients and people with consciousness disorders recommend indwelling gastric tube.

Admission hyperglycemia was diagnosed when the random blood glucose level on admission was ≥11.1 mmol/L, while fasting hyperglycemia was diagnosed when the blood glucose level at the next morning after admission was ≥7.1 mmol/L.

SAP was diagnosed according to the modified Centers for Disease Control and Prevention (CDC) criteria [2] and/or Mann’s criteria [17]. All the patients included in this study were diagnosed with SAP by the Mann’s criteria and the modified CDC criteria. Definitive SAP was recorded as having SAP. SAP is diagnosed as long as one of the criteria is met.

The assignment rules for the independent risk factor of SAP were as follows: 1 point was assigned for 1.25 < adjusted odds ratio (OR) < 2.0; 2 points were assigned for 2.0 ≤ adjusted OR < 4.0; 3 points were assigned for 4.0 ≤ adjusted OR < 6.0 [7].

### Statistical analyses

SPSS 20.0 software (IBM Corp., Armonk, NY, USA) and MedCalc 15.2.2 software (MedCalc Software bvba) were used for the statistical analyses.

The following analysis used SPSS 20.0 software. Continuous variables were expressed as the mean ± standard deviation (SD). Categorical variables were expressed as percentage. Continuous variables were compared using nonparametric tests, and categorical variables were compared using Chi-squared test. Logistic regression was used to calculate the adjusted OR value after balancing the confounding factors. Hyperglycemia was assigned according to the above assignment rules, and the a modified A^2^DS^2^ scoring system was designed by combining the conventional A^2^DS^2^ items and assigned hyperglycemia points.

Receiver operating characteristic (ROC) curve analysis method in MedCalc 15.2.2 software was used to calculate the area under the curve of the conventional A^2^DS^2^ scoring system and modified A^2^DS^2^ scoring system. The cut-off values that represented the sensitivity and specificity of each evaluation tool were analyzed. Points with the maximal Youden’s index were obtained as the optimal cut-off values. The ROC curve comparison method was used to compare whether the improved A2DS2 score was superior to the A2DS2 score.

Probability (*p*) values ≤0.05 were considered statistically significant.

## Results

In strict accordance with the inclusion and exclusion criteria set in this study, a total of 2552 patients were included in this study. The average age of the enrolled patients was 61.9 ± 12.7 years. SAP were observed in 7.8% (200 cases) of all patients. There was no significant difference in gender and infarction site between the SAP group and non-SAP group (*P* > 0.05). The incidence of SAP in patients with the following factor(s) was significantly higher than that in patients without the following factor(s) (*P <* 0.05): elderly age (≥75 years old), atrial fibrillation, dysphagia, random hyperglycemia, fasting hyperglycemia, or random or fasting hyperglycemia. Therefore, these factors were considered as potential risk factors for SAP. These patients were classified into three subgroups according to stroke severity (NIHSS score), and there was a statistically significant difference in the incidence of pneumonia among these three subgroups (*P* < 0.05). The clinical characteristics of patients in the SAP group and non-SAP group, and the relevant statistical results are summarized in Table 1.

**Table 1:** Statistical analysis of clinical characteristics between two groups

Variables	Non-SAP	SAP	χ^2^	OR	*P* value
Elderly age (≥75 years old)	396 (16.8%)	87 (43.5%)	85.402	3.803	< 0.001
Male sex	1635 (69.5%)	133 (66.5%)	0.787	1.148	0.375
Atrial fibrillation	121 (5.1%)	50 (25.0%)	116.239	6.146	< 0.001
Dysphagia	389 (16.5%)	51 (25.5%)	10.373	1.727	0.001
NIHSS score (≥16 points)*	12 (0.5%)	33 (16.5%)	–	–	< 0.001
NIHSS score (5–15 points)*	742 (31.5%)	114 (57.0%)	–	–	< 0.001
NIHSS score (0–4 points)*	1598 (67.9%)	53 (26.5%)	–	–	< 0.001
Random hyperglycemia	257 (10.9%)	35 (17.5%)	7.860	1.729	0.005
Fasting hyperglycemia	566 (24.1%)	95 (47.5%)	52.747	2.855	< 0.001
Random or fasting hyperglycemia	619 (26.3%)	99 (49.5%)	48.992	2.744	< 0.001
Posterior circulation infarction	772 (32.8%)	71 (35.5%)	0.597	1.126	0.440

*NIHSS*, National Institutes of Health Stroke Scale

*NIHSS scores was compared using nonparametric tests

There was no significant difference in gender and infarction site between the SAP group and non-SAP group (*P* > 0.05). The incidence of SAP in patients with the following factor(s) was significantly higher than that in patients without the following factor(s) (*P <* 0.05): elderly age (≥75 years old), atrial fibrillation, dysphagia, random hyperglycemia, fasting hyperglycemia, or random or fasting hyperglycemia. Therefore, these factors were considered as potential risk factors for SAP. These patients were classified into three subgroups according to stroke severity (NIHSS score), and there was a statistically significant difference in the incidence of pneumonia among these three subgroups (*P* < 0.05)

Logistic regression analysis was further used to calculate the adjusted OR value of each potential risk factor. The logistic regression analysis results revealed that elderly age (≥75 years old), male gender, atrial fibrillation, dysphagia, an NIHSS score of 5–15 points, NIHSS of ≥16 points, and fasting hyperglycemia were independent risk factors of SAP (*P* < 0.05). The detailed statistical results are prsented in Table 2. The adjusted OR value of fasting hyperglycemia was 2.95, which was assigned with 2 points in the modified A^2^DS^2^ scoring system (Table 3).

**Table 2:** Stepwise logistic regression analysis showing independent risk factors of stroke-associated pneumonia

Risk factors	Adjusted OR	95% confidence interval	*P* value
Elderly age (≥75 years old)	3.94	2.77–5.62	< 0.001
Male sex	1.61	1.12–2.33	0.011
Atrial fibrillation	3.33	2.11–5.25	< 0.001
Dysphagia	1.74	1.19–2.54	0.004
NIHSS score (5–15 points)	3.95	2.77–5.62	< 0.001
NIHSS score (≥16 points)	91.64	42.04–199.75	< 0.001
Fasting hyperglycemia	2.95	2.11–4.12	< 0.001

*NIHSS*, National Institutes of Health Stroke Scale

The logistic regression analysis results revealed that elderly age (≥75 years old), male gender, atrial fibrillation, dysphagia, an NIHSS score of 5–15 points, NIHSS of ≥16 points, and fasting hyperglycemia were independent risk factors of SAP (*P* < 0.05)

**Table 3:** Modified A^2^DS^2^ score (combined conventional A^2^DS^2^ score and fasting hyperglycemia)

Clinical characteristics on admission	Assigned points
Elderly age (≥75 years old)	+ 1
Male sex	+ 1
Atrial fibrillation	+ 1
Dysphagia	+ 2
Stroke severity	
NIHSS score (5–15 points)	+ 3
NIHSS score (≥16 points)	+ 5
Fasting hyperglycemia	+ 2

*NIHSS*, National Institutes of Health Stroke Scale

The adjusted OR value of fasting hyperglycemia was 2.95, which was assigned with 2 points in the modified A^2^DS^2^ scoring system

**Fig. 1:**
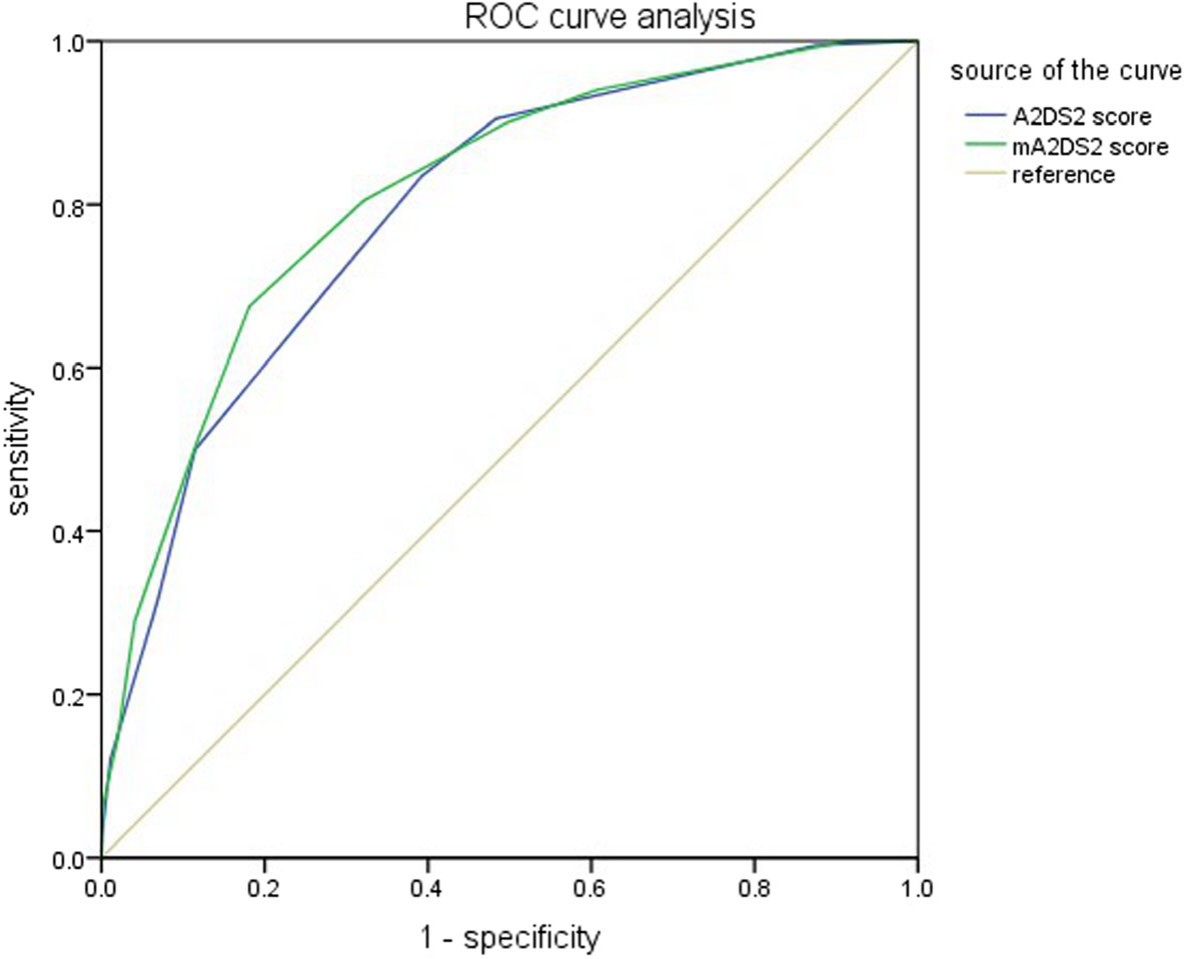
ROC curve analysis

ROC curve analysis was used to evaluate the sensitivity and specificity of each cut-off value. The statistical results are presented in Table 4, while the ROC curves are presented in Fig. 1. When the cut-off value was 4 points, the sensitivity and the specificity were 80.5 and 67.9%, respectively. Hence, 4 points can be used as the optimal operating point of the modified A^2^DS^2^ scoring system.

**Table 4:** Cut-off values and corresponding sensitivity and specificity

Cut-off value	Modified A^2^DS^2^ score			Conventional A^2^DS^2^ score		
	Sensitivity (%)	Specificity (%)	Youden index	Sensitivity (%)	Specificity (%)	Youden index
1	100.0	8.5	0.085	99.5	12.5	0.12
2	94.0	39.4	0.334	90.5	51.7	0.422
3	90.0	50.4	0.404	83.5	60.8	0.443
4	80.5	67.9	0.484	69.0	72.9	0.419
5	67.5	81.9	0.494	50.0	88.5	0.385
6	51.0	88.3	0.393	31.5	93.1	0.246
7	29.0	95.9	0.249	12.0	98.9	0.109
8	14.5	98.0	0.125	3.0	99.9	0.029
9	6.5	99.8	0.063	0.5	100.0	0.005
10	1.5	100.0	0.015	0.0	100.0	0

When the cut-off value was 4 points, the sensitivity and the specificity were 80.5 and 67.9%, respectively

The area under curve of the modified A^2^DS^2^ scoring system was significantly higher than that of the conventional A^2^DS^2^ scoring system (*F* test, *P* < 0.05; Table 5 and Fig. 1), indicating the modified A^2^DS^2^ scoring system had higher predictive efficiency.

**Table 5:** Area under curve

Scoring system	Area under curve	Standard Error	*Z* value	*P* value
Modified A^2^DS^2^ score	0.814	0.0153	2.336	0.020
Conventional A^2^DS^2^ score	0.793	0.0155		

The area under curve of the modified A^2^DS^2^ scoring system was significantly higher than that of the conventional A^2^DS^2^ scoring system

## Discussion

In the present study, the value of the modified A^2^DS^2^ scoring system (combining the conventional A^2^DS^2^ scoring system with hyperglycemia) for predicting SAP was investigated. It was found that fasting hyperglycemia is an independent risk factor of SAP, which may be a valuable indicator for predicting SAP. Moreover, the predictive value of the modified A^2^DS^2^ scoring system is better than that of the conventional A^2^DS^2^ scoring system.

In present clinical practice, evaluating the risk of SAP remains challenging. The A^2^DS^2^ scoring system, in which scoring items include age, gender, atrial fibrillation, dysphagia and severity of stroke, has been proven to be a simple and reliable scoring scale. However, in literature, hyperglycemia has also been reported as a potential risk factor of SAP, although the evidence remains controversial. Hoffman et al. found that the history of diabetes was not an independent risk factor for SAP [7] . It is noteworthy that the history of diabetes cannot represent an abnormal blood glucose level, and temporary hyperglycemia may indicate stress hyperglycemia, rather than diabetes. In addition, diabetic patients with poor blood glucose control would most likely suffer from cerebral infarction. Thus, the correlation between hyperglycemia and SAP, as well as the value of hyperglycemia for predicting SAP, were investigated.

According to the international diagnosis and treatment guidelines for acute stroke, blood glucose level is recommended as a routine screening index for all patients. In the present study, the random blood glucose level after the onset of stroke represented stress hyperglycemia. Fasting hyperglycemia represented diabetes with poor glycemic control or newly-onset diabetes. Random or fasting hyperglycemia represented an increased blood glucose level caused by different causes. After statistical comparisons, merely fasting hyperglycemia entered the logistic regression model. It was speculated that stress hyperglycemia may be not an independent risk factor for SAP, and occasional transient hyperglycemia cannot increase the risk of SAP. Patients with fasting hyperglycemia (diabetes with poor glycemic control or newly-onset diabetes) are more likely to have SAP, which is consistent with previous findings [6, 14, 15] .

The present study indicated that fasting hyperglycemia is an independent risk factor for SAP. Acute ischemic stroke can cause hyperglycemia mainly through the following mechanisms: the activation of the sympathetic and parasympathetic nervous system [18–20], and the immune response of the hypothalamic-pituitary-adrenal axis [19–21]. Hyperglycemia can reduce the bactericidal ability of leukocytes, increasing the likelihood of pulmonary infection [22, 23] . The study conducted by Obiako et al. revealed that the proportion of hyperglycemia was greater than that of diabetes in patients with acute stroke [24], suggesting that the poor prognosis of acute stroke may be attributed to hyperglycemia induced by stress reaction, rather than diabetes.

A number of studies have shown that hyperglycemia is significantly correlated with the occurrence of pneumonia and the poor outcome of acute ischemic stroke, especially in patients without diabetes. Dziedzic et al. noted that the incidence of pneumonia was higher in non-diabetic patients with fasting hyperglycemia. Nevertheless, the multivariate analysis revealed that fasting hyperglycemia was not significantly associated with pneumonia [25]. Hirata et al. reported that the mortality of pneumonia was significantly correlated with hyperglycemia during hospitalization, but was not correlated to the history of diabetes [26]. It was speculated that hyperglycemia may be associated with the severity and poor prognosis of acute stroke in non-diabetic patients, and diabetic patients may have adapted to the long-term hyperglycemia, which can protect the brain tissue against acute blood glucose increase.

The management of hyperglycemia should be highlighted during hospitalization for reducing the risks of SAP. Blood glucose level is correlated to the functions of various intracranial systems, such as the cerebrovascular system, inflammatory system, and metabolic system [27]. Appropriate blood glucose control can improve immunosuppression and decrease the incidence and severity of infection. The optimal treatment of hyperglycemia in patients with acute stroke remains to be well-elucidated.

Furthermore, the average age of patients in the present study was lower than that reported in the study conducted by Hoffman et al. (61.9 ± 12.7 vs. 71.2 ± 13.1) [7]. This discrepancy may indicate a different age distribution between China and Germany. ROC curve analysis has been widely used for making the best diagnostic criteria, and determining the best critical value, while the area under curve can represent the efficiency of the prediction. In the present study, the area under curve of the modified A^2^DS^2^ scoring system was significantly higher than that of the conventional A^2^DS^2^ scoring system, suggesting that the modified system (including the item of hyperglycemia) is more effective for predicting SAP. This modified A2DS2 scoring system may help to predict the risk of SAP in early stage of stroke patients more effectively, allowing timely prophylactic treatment, such as antibiotic therapy and the prophylactic use of aspiration.

The present study has some strengths. At present, reliable tools for predicting the risk of SAP include the A^2^DS^2^ scoring system and AIS-APS scale [6, 7]. In the present study, the former one was utilized, since it was more simple and practical. In addition, the inclusion and exclusion criteria were strict in the present study, and all researchers were uniformly trained [2] . The novelty of the present study was the combination of the A^2^DS^2^ scoring system and evaluation of hyperglycemia. The present findings may improve the predictive value of the A^2^DS^2^ scoring system.

There were still some limitations in the present study. First, the dynamic changes of the blood glucose levels of patients were not monitored throughout hospitalization, and only the random blood glucose level on admission was assessed. Second, the single-center and retrospective design was an inherent defect of the present study. In the future research, more external verifications are needed to arrive at a definitive conclusion.

## Conclusion

Fasting hyperglycemia is an independent risk factor for predicting SAP. Compared with the A2DS2 score, the modified A2DS2 score (combined A^2^DS^2^ score and fasting hyperglycemia) is more effective in predicting the risk of SAP.

## Supplementary information

**Additional file 1.** Supplementary material

## Abbreviations

CDC: The Centers for Disease Control and Prevention criteria
MRI: Magnetic resonance imaging
NIHSS: National Institute of Health stroke scale
OR: odds ratio
ROC: Receiver operating characteristic
SAP: Stroke-associated pneumonia
SD: Standard deviation
TIA: Transient ischemic attack
